# The Australian Defence Force Mental Health Prevalence and Wellbeing Study: design and methods

**DOI:** 10.3402/ejpt.v5.23950

**Published:** 2014-08-14

**Authors:** Miranda Van Hooff, Alexander C. McFarlane, Christopher E. Davies, Amelia K. Searle, A. Kate Fairweather-Schmidt, Alan Verhagen, Helen Benassi, Stephanie E. Hodson

**Affiliations:** 1 Centre for Traumatic Stress Studies, The University of Adelaide, South Australia; 2 Data Management and Analysis Centre, Discipline of Public Health, The University of Adelaide, South Australia; 3 School of Psychology, Flinders University, South Australia; 4 Mental Health, Psychology and Rehabilitation Branch, Joint Health Command, Department of Defence, Canberra, Australian Capital Territory, Australia; 5 Department of Veterans' Affairs, Canberra, Australian Capital Territory, Australia

**Keywords:** Prevalence, military, mental disorder, affective, anxiety, alcohol

## Abstract

**Background:**

The Australian Defence Force (ADF) Mental Health Prevalence and Wellbeing Study (MHPWS) is the first study of mental disorder prevalence in an entire military population.

**Objective:**

The MHPWS aims to establish mental disorder prevalence, refine current ADF mental health screening methods, and identify specific occupational factors that influence mental health. This paper describes the design, sampling strategies, and methodology used in this study.

**Method:**

At Phase 1, approximately half of all regular Navy, Army, and Air Force personnel (*n*=24,481) completed self-report questionnaires. At Phase 2, a stratified sub-sample (*n*=1,798) completed a structured diagnostic interview to detect mental disorder. Based on data from non-responders, data were weighted to represent the entire ADF population (*n*=50,049).

**Results:**

One in five ADF members met criteria for a 12-month mental disorder (22%). The most common disorder category was anxiety disorders (14.8%), followed by affective (9.5%) and alcohol disorders (5.2%). At risk ADF sub-groups were Army personnel, and those in the lower ranks. Deployment status did not have an impact on mental disorder rates.

**Conclusion:**

This study has important implications for mental health service delivery for Australian and international military personnel as well as contemporary veterans.

Military service is an occupation where personnel are selected and trained to face stressful and potentially traumatising situations. The ways in which military personnel respond to and adapt to military service and particularly combat environments have considerable relevance to the design of preventive programs, the provision of treatment, and projecting the long-term needs of veterans.

To date, the major focus of research investigating mental health among military populations has been to report on particular sub-samples of interest, rather than making an estimate of the prevalence rates in an entire force. In particular, the studies to date have been of single services or battalions, treatment-seeking samples, and cohorts from specific deployments representing only a proportions of total defence populations from which the sample is drawn (Bachynski et al., 2012; Hoge et al., 2004; Ismail et al., 2002; Jones et al., 2008). Furthermore, most studies are predominantly reliant upon self-report surveys that bring the attended limitations of their validity compared to structured interviews (Pinder et al., 2012).

An important exception from a sampling perspective is the Millennium Cohort study that aimed to recruit a representative sample of US military personnel (Riddle et al., 2007; Ryan et al., 2007). This study surveyed 77,047 US military personnel across all services; 3.5% of the 2.2 million US armed forces personnel in service in 2000, using the self-report Primary Care Evaluation of Mental Disorders Patient Health Questionnaire (PHQ) and the post-traumatic stress disorder (PTSD) Checklist—Civilian Version (PCL-C). In this study 18.3% met criteria for a mental disorder, 12.6% reported alcohol abuse, 3.2% reported a major depressive disorder, and 1.0% reported panic syndrome (Riddle et al., 2007). PTSD rates were higher in deployed personnel exposed to combat (7.6%) compared to deployed personnel without combat (1.4%) and personnel who had not deployed (2.3%) (Pinder et al., 2012; Smith et al., 2008).

The King's Cohort, another important large scale cohort study of 10,272 British armed forces personnel and veterans (3–4% of the total UK armed forces) was designed for a different purpose, namely to capture the impact of Op TELIC in Iraq. The comparison group was stratified to match those deployed to Iraq rather than the British military more generally, and excluded Special Force and high security personnel. Although this sample gave a substantial picture of the UK military, including reservists, it was not a representative sample of the entire defence force.

The first wave recorded a similar prevalence of 20% for the presence of a common mental disorder using the 12-item self-report General Health Questionnaire (GHQ-12) and 4% for PTSD using the PCL-C (Hotopf et al., 2006). Additional assessment using the PHQ, and the 4-item Primary Care PTSD (PC-PTSD) administered via interview resulted in disorder rates of 28.9% overall, 4.8% with probable PTSD, 3.7% with major depressive syndrome, and 18% with alcohol abuse, with PTSD being relatively consistent between those who had deployed and never deployed (Iversen et al., 2009; Jones et al., 2013). A second wave of participants was recruited to make the sample more representative of the military but had more army and those likely to be deployed on operations (Fear et al., 2010). Studies of this genre have been critical in answering the questions about the specific impact of these recent conflicts, using carefully constructed comparison groups.

To date, only one published epidemiological military study has specifically focussed on making prevalence estimates of an entire defence force using a structured diagnostic interview (Sareen et al., 2007). Specifically, a stratified sample of 5,154 regular Canadian Defence Force members completed the Composite International Diagnostic Interview (CIDI) version 2.1. This study demonstrated a prevalence of any 12-month mental disorder of 14.9%. However, due to its single-phase design, this study is limited with respect to prevalence rate estimation and case detection, particularly for low prevalence disorders.

This paper describes the Australian Defence Force (ADF) Mental Health Prevalence and Wellbeing Study (MHPWS) (McFarlane, Hodson, Van Hooff, & Davies, 2011) that was initiated in response to recommendations made in the 2009 Review of Mental Health Care in the ADF and Transition Through Discharge (Dunt, 2009). It had three primary aims: to 1) provide prevalence estimates of 12-month and lifetime mental disorder in currently serving regular ADF personnel using a two-phase design and a gold standard psychiatric interview; 2) refine current ADF mental health detection methods by establishing valid cut-offs for three post-deployment psychological screening instruments; and 3) investigate specific occupational factors (i.e., ADF service, deployment, support networks, help-seeking, and barriers to care) that influence mental health in the ADF.

The ADF comprises regular and reserve personnel in the three services of Navy, Army and Air Force. In 2010, ADF personnel were deployed to locations including Afghanistan, Iraq, East Timor, and Solomon Islands, as well as contributing to the United Nations and other peacekeeping operations worldwide with 62% having been deployed to one or more location at some stage of their career. At the time of the study in 2010, just over one third (*N=*18,625, 37%) of regular ADF members had deployed to the Middle East Area of Operations (MEAO).

The MHPWS study was established to make the first comprehensive assessment of the current mental health status of personnel in the ADF, and to provide a baseline for future longitudinal health surveillance, in the context of the recent war-like deployments to Iraq and Afghanistan. It aimed to compare the impact of non-deployed and deployed service, and to appraise the validity and usefulness of current ADF screening tools. Unlike previous studies of military populations, this study used a two-phase design in combination with a diagnostic interview [WMH CIDI version 3·0 (Kessler & Üstün, 2004)] to obtain prevalence estimates of 12-month ICD-10 mental disorder in a defence force, excluding reservists. This paper describes the MHPWS study methodology, the demographic characteristics of ADF personnel, and provides an overview of mental disorder prevalence.

## Materials and methods

### Study participants

**Fig. 1:**
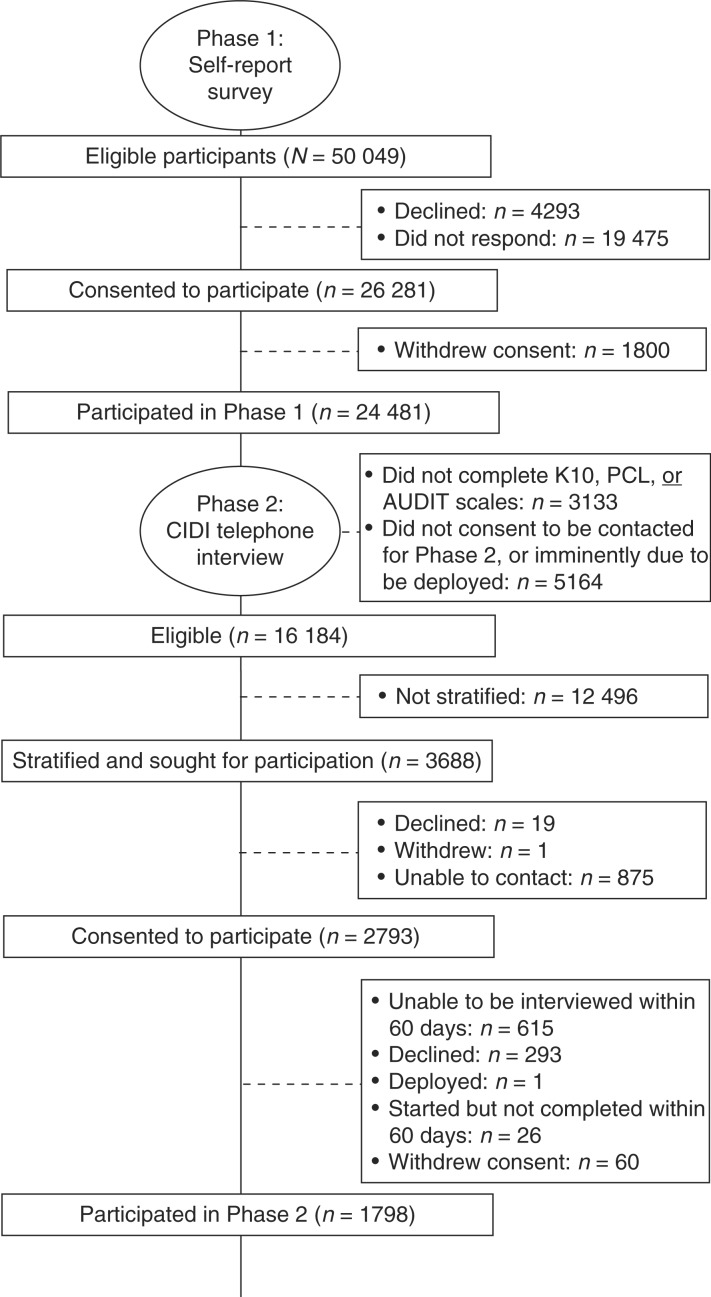
Flow chart of the progression of participants through the study.

All recruitment and assessment were conducted between April 2010 and January 2011. The flow of participants through the study phases is shown in Fig. 1. At completion, 52.5% (26,281) of ADF personnel had consented to participate in the study, 8.6% (4,293) declined to participate, and 38.9% (19,475) did not respond. Although Defence provided the research team with the contact details for all ADF personnel, the responses to this survey and interview were de-identified and participants were informed that no personal details, including whether or not they participated in the study would be provided to Defence or the Department of Veteran's Affairs (DVA).

### Phase 1—self-report questionnaire

At Phase 1, all currently-serving^1^ regular ADF personnel in the Navy, Army, and Air Force (excluding trainees and reservists) were invited to participate (*n*=50,049). Members’ contact details were obtained from defence records. The study was advertised through defence-based media, forums, and introductory letters were sent to individual participants. Initial contact (including consent form and questionnaire distribution) was via email and mail. Subsequent email and mail reminders, Defence base visits and finally telephone calls to non-respondents were used to maximise the return rates. In total, 24,481 ADF members participated (49% of the ADF) by completing self-report questionnaires.

### Phase 2—CIDI interview

At Phase 2, a stratified sub-sample of 3,688 (15% of the Phase 1 sample) were selected to complete a telephone diagnostic interview, of which, 1,798 (49%) participated. In total, 87.5% of CIDI interviews were completed within 60 days of the self-report booklet, with 35.6% (640) completing the interview within 28 days. The mean number of days between completion of the self-report survey and the CIDI interview was 42.0 (SD=25.3).

A maximum of 10 attempts were made to speak to participants before they were removed from the participant pool. Informed consent was digitally recorded over the telephone. Interviewers were blind to participants’ screening questionnaire scores.

### Design

The two-phase design (Pickles, Dunn, & Vazquez-Barquero, 1995) of this epidemiological study is well accepted for investigating mental disorder prevalence (Salim & Welsh, 2009) because of the potential increased efficiency with respect to the estimation of prevalence rates and case detection (Newman, Shrout, & Bland, 1990). Specifically:

- Phase 1 investigated levels of psychological and physical symptoms through a self-report questionnaire, which is economical of time and resources.
- Phase 2 examined mental disorder prevalence within the ADF. To do this, a stratified sub-sample of Phase 1 respondents was selected to complete a more accurate but costly structured diagnostic interview—the CIDI.

The study protocol was approval by the Defence and University human ethics committees.

### Stratification

Phase 2 selection for the CIDI interviews was based on a stratification procedure involving four variables: service, sex, and specific Phase 1 psychological questionnaire scores—the PCL-C (Weathers, Litz, Herman, Huska, & Keane, 1994), and Alcohol Use Disorders Identification Test (Saunders, Aasland, Babor, De la Fuente, & Grant, 1993) (AUDIT). The decision to use these questionnaires was based on previous surveys of ADF members who had been deployed to the Near North Area of Influence (McGuire et al., 2009a, b).

The 60th and 80th percentiles of the PCL and AUDIT distributions were used as cut-offs to form three stratification bands:

- Band 1: PCL≤25 and AUDIT≤7
- Band 2: (25<PCL≤33 and AUDIT≤10) or (PCL≤33 and 7<AUDIT≤10)
- Band 3: PCL>33 or AUDIT>10

Using these bands, the higher-scoring ADF members were oversampled to provide adequate power to make more accurate prevalence estimates (those in Bands 2 and 3).

In addition to these questionnaires, sex (oversampling for females) and service (oversampling for Navy and Air Force personnel) were used to select participants, to account for the greater number of males and Army personnel in the ADF, to ensure appropriate weighting.

Table 1 shows the numbers of participants selected for an interview in each stratum.

**Table 1:** Phase 2 interview strata sampling numbers and percentages (*n*=3,688)

	Female, *n* (%)	Male Navy, *n* (%)	Male Army, *n* (%)	Male Air Force, *n* (%)
Band 3 (high scorers)	192 (100)	260 (100)	690 (100)	297 (100)
Band 2	263 (100)	155 (50)	174 (20)	313 (30)
Band 1 (low scorers)	452 (50)	195 (20)	139 (5)	558 (10)

Band 1: PCL≤25 and AUDIT≤7; Band 2: 25<PCL≤33 and AUDIT≤10, or PCL≤33 and 7<AUDIT≤10; Band 3: PCL>33 or AUDIT>10.

### Weighting

Weights were applied to both the questionnaire and interview data to provide ADF population prevalence estimates. To develop weights, demographic information (e.g., sex, service, rank) was obtained from the ADF nominal roll.

### Phase 1

To correct for differential non-response, questionnaire results were weighted based on strata derived from sex, service, rank, and medical employment classification (MEC) status. Within each stratum the weight was calculated as the population size divided by the number of respondents from the stratum. In each questionnaire section, responses were only used if the participant responded to all of the questions from that section. Thus, separate weights were calculated for each section. A finite population correction was also applied to adjust the variance estimates for the reasonably large sampling fraction within each stratum.

### Phase 2

Within each stratum the weight was calculated as the population size divided by the number of interview respondents from the stratum. As band was not available for non-responders, the population size within each stratum was estimated by multiplying the known sex by service population total by the observed proportion belonging to the band of interest from within the corresponding stratum. A finite population correction was also applied to adjust the interview variance estimates.

When outputs by sex, service, and rank were required, post-stratification by these variables was used to adjust the weights so that known population totals were reproduced by the estimates. This also accounted for the known differential non-response by rank to the survey.

### Measures

#### Baseline Phase 1: self-report questionnaire booklet

The questionnaire booklet comprised a series of standard demographic questions, as well as several physical and psychological health measures. To screen for potential mental health problems, the same psychological screening instruments utilised by the ADF post-deployment were used. These measures included the AUDIT (Babor, Higgins-Biddle, & Saunders, 2001; Saunders et al., 1993) to establish hazardous and harmful drinking patterns; the PCL-C (Weathers et al., 1994) to identify post-traumatic stress symptoms; and the Kessler Psychological Distress Scale—10-item version (K10) (Kessler et al., 2002) to assess psychological distress. These measures are widely used in civilian, military, and veteran research, and have strong psychometric properties. The PCL, for example, has shown good overall diagnostic accuracy in various primary care and veteran samples (McDonald & Calhoun, 2010; Wilkins, Lang, & Norman, 2011). The AUDIT shows high sensitivity in primary care patients and epidemiological populations, with slightly lower, though acceptable specificity (Reinert & Allen, 2002). The Kessler Psychological Distress Scale (10-item version) shows high levels of overall diagnostic accuracy and excellent psychometric properties in numerous studies (Andrews & Slade, 2001; Furukawa, Kessler, Slade, & Andrews, 2003; Kessler & Üstün, 2004; Kessler et al., 2002; Oakley Browne, Wells, Scott, & McGee, 2010). A full description of this survey and its constituent questions can be found in the MHPWS report (McFarlane et al., 2011), including the documentation of deployment history. War-like deployment was defined by specific criteria used for determinations under the Veterans Entitlement Act.

#### Phase 2: structured diagnostic interview

Phase 2 participants were administered the CIDI version 3 (World Health Organization Computer Assisted Psychiatric Interview) (Kessler & Üstün, 2004) via telephone. Twelve-month and lifetime ICD-10 rates of the following disorders were assessed: depressive episode, dysthymia, bipolar affective disorder, panic attack, panic disorder, agoraphobia, social phobia, specific phobia, generalised anxiety disorder, obsessive-compulsive disorder, PTSD, alcohol harmful use, and alcohol dependence. Clinical calibration studies report the CIDI version 3 to have good validity in civilian populations (Haro et al., 2006).

Throughout this report, the ICD-10 prevalence rates are presented with hierarchy rules applied. For all ICD-10 disorders, the standard WMH–CIDI 3.0 algorithms were applied, which means that in order for a 12-month diagnosis to be given, an individual would be required to meet lifetime criteria initially and then have reported symptoms in the 12 months prior to the interview. The interview was administered by interviewers who underwent accredited CIDI training, all of whom had a minimum qualification of an honours degree in psychology. Recorded interviews were monitored weekly for quality training purposes at the Centre for Traumatic Stress Studies (CTSS).

The mental health prevalence estimates provided in this paper are based on the Phase 2 interview data. The associated demographic predictors such as sex, service, rank, and deployment are described. Four broad categories of 12-month disorders are reported here: 1) any affective disorder: a 12-month ICD-10 diagnosis of mild, moderate, or severe depression; dysthymia; or bipolar affective disorder; 2) any anxiety disorder: a 12-month ICD-10 diagnosis of panic disorder, panic attacks, agoraphobia, simple phobia, social phobia, generalised anxiety disorder, obsessive-compulsive disorder or PTSD; 3) any alcohol disorder: a 12-month ICD-10 diagnosis of alcohol harmful use or alcohol dependence, and 4) any mental disorder: 12-month ICD-10 diagnosis of an affective, anxiety, or alcohol disorder.

#### Statistical analysis

Analyses were conducted in Stata version 11.2 or SAS version 9.2. All analyses were conducted using weighted estimates of totals, means, and proportions. Standard errors were estimated using linearisation.

Predictors of the four ICD-10 disorder groups were analysed using simultaneous multivariate logistic regression. Predictors were sex, rank, service, and deployment status (never deployed, deployed). The interaction between sex and service was initially included, but was removed if found to be non-significant. No other interactions were included.

## Results

### Sample and population characteristics

The breakdown of individuals with enough data to be included in the survey analysis sample is summarised in Table 2.

**Table 2:** Phase 1 survey response rates by service for the Mental Health Prevalence and Wellbeing Study

	Population	Responders	Rate (%)
Total ADF	50,049	24,481	48.9
Navy	11,612	5,392	46.4
Army	25,356	11,429	45.1
Air Force	13,081	7,760	59.3

As the population characteristics were known (i.e., gender, service, MEC status, and deployment history), it was possible to compare personnel who responded to the survey with the target population. Sample demographic characteristics of the two data collection phases and their target populations are shown in Table 3. Although slight differences between the samples and their target populations were found, these observed differences were subsequently used in the population weighting process; therefore any resultant estimates generated effectively represented the entire ADF population.

**Table 3:** Demographic profile of Phase 1 survey and Phase 2 interview responders, and target populations

	Phase 1: Survey				Phase 2: CIDI interview			
		
	Target: ADF population (*N*=50,049)		Survey responders (*n*=24,481)		Target: selected for CIDI (*N*=3,688)		CIDI responders (*n*=1,798)	
				
Characteristic	*n*	%	*n*	%	*n*	%	*n*	%
Sex								
Female	6,808	13.6	3,888	15.9	907	24.6	438	24.4
Male	43,241	86.4	20,593	84.1	2,781	75.4	1,360	75.6
Service								
Navy	11,612	23.2	5,392	22.0	837	22.7	384	21.4
Female	2,104	4.2	1,053	4.3	227	6.2	100	5.6
Male	9,508	19.0	4,339	17.7	610	16.5	284	15.8
Army	25,356	50.7	11,429	46.7	1,325	35.9	716	39.8
Female	2,513	5.0	1,437	5.9	322	8.7	165	9.2
Male	22,843	45.6	9,992	40.8	1,003	27.2	551	30.6
Air Force	13,081	26.1	7,660	31.3	1,526	41.4	698	38.8
Female	2,191	4.4	1,398	5.7	358	9.7	173	9.6
Male	10,890	21.8	6,262	25.6	1,168	31.7	525	29.2
Age (years)	33.2 (M)	9.2 (SD)	35.5 (M)	9.3 (SD)	37.3 (M)	9.4 (SD)	38.3 (M)	9.4 (SD)
Marital status								
Married	31,500	62.9	18,882	77.1	2,862	77.6	1,388	77.2
Not married	18,549	37.1	5,599	22.9	826	22.4	410	22.8
Education								
Missing	–a	–a	396	1.6	4	0.1	1	0.1
High school or less	–a	–a	2,888	11.8	449	12.2	235	13.1
Certificate/diploma	–a	–a	8,755	35.8	1,390	37.7	663	36.9
Bachelor degree	–a	–a	3,119	12.7	560	15.2	298	16.6
Post-graduate	–a	–a	9,323	38.1	1,285	34.8	601	33.4
Rank								
Commissioned officer	12,034	24.0	7,268	29.7	1,233	33.4	655	36.4
Non-commissioned officer	22,319	44.6	12,381	50.6	1,881	51.0	889	49.4
Other ranks	15,696	31.4	4,832	19.7	574	15.6	254	14.1
MEC status								
MEC 1	32,816	65.6	14,954	61.1	1,989	53.9	906	50.4
MEC 2	11,712	23.4	6,726	27.5	1,184	32.1	611	34.0
MEC 3	4,485	9.0	2,301	9.4	413	11.2	224	12.5
MEC 4	1,036	2.1	500	2.0	102	2.8	57	3.2
ADF deployment								
Missing	983	2.0	0	0.0	0	0.0	0	0.0
Yes	16,986	33.9	15,952	65.2	2,288	62.0	1,111	61.8
No	32,080	64.1	8,529	34.8	1,400	38.0	687	38.2
Length of service in the ADF (years)	11.6 (M)	8.8 (SD)	13.7 (M)	9.3 (SD)	15.3 (M)	9.5 (SD)	16.2 (M)	9.8 (SD)

MEC=Medical Employment Classification (smaller classification numbers indicate greater medical fitness for deployment).

a These data could not be obtained for non-responders.

Compared to Phase 1 non-responders, Phase 1 responders were more likely to be female, in the Air Force, slightly higher in age, married, and non-commissioned officers. ADF personnel who were MEC 2 (27.5%) and MEC 3 (9.4%) were slightly overrepresented in the responders. In contrast, deployment and education had little impact on the response rates.

Phase 2 responders were more likely to be in the Army, to be older, to be officers, and to have a medical classification of 2, 3, or 4. Gender, marital status, deployment status, and length of service in the ADF had little impact on the response rates for the CIDI interview.

### Mental disorder prevalence

Baseline prevalence data obtained using the CIDI (Table 4) demonstrates that in the 12 months prior to the interview one in five ADF members met criteria for a mental disorder (22%, 95% CI 19.9, 25.2). The most common disorder category was anxiety disorders (14.8%, 95% CI 11.9, 17.1) followed by affective disorders (9.5%, 95% CI 7.2, 11.8) and alcohol disorders (5.2%, 95% CI 3.8, 6.6). Although not the main focus of this report, the 30-day prevalence rates were: for any anxiety disorder 7.5% (95% CI 5.4, 9.7), for any affective disorder 2.6% (95% CI 1.9, 3.4), and for any alcohol disorder 1.0% (95% CI 0.5, 1.4).

**Table 4:** Weighted prevalence (w%) of 12-month ICD-10 disorder among Australian Defence Force Personnel

	Any affective disorder		Any anxiety disorder		Any alcohol disorder		Any mental disorder	
				
Characteristic	*w*%	95% CI	*w*%	95% CI	*w*%	95% CI	*w*%	95% CI
Full cohort	9.5	7.2–11.8	14.8	11.9–17.7	5.2	3.8–6.6	22.0	18.9–25.2
Sex								
Female	10.2	7.5–12.9	18.8	15.0–22.5	2.2	0.9–3.6	24.1	20.0–28.2
Male	9.4	6.8–12.0	14.2	10.9–17.5	5.6	4.1–7.2	21.7	18.1–25.3
Service								
Navy	10.5	7.1–13.9	14.1	10.7–17.6	7.6	3.8–11.4	24.5	19.4–29.6
Female	12.9	6.1–19.8	18.5	10.5–26.5	3.7	0.1–7.3	26.5	17.2–35.7
Male	10.0	6.1–13.9	13.1	9.3–17.0	8.5	3.9–13.0	24.1	18.2–30.0
Army	10.6	6.4–14.8	17.3	11.8–22.7	5.6	3.6–7.6	24.4	18.8–30.1
Female	9.2	5.8–12.6	18.9	18.9	1.5	0.0–3.2	23.0	16.5–29.6
Male	10.8	6.1–15.4	17.1	11.1–23.1	6.0	3.8–8.3	24.6	18.4–30.8
Air Force	6.4	4.8–8.1	10.7	8.8–12.7	2.2	1.1–3.3	15.1	12.7–17.5
Female	8.8	5.4–12.1	19.0	14.0–23.9	1.6	0.0–3.2	23.1	17.7–28.4
Male	6.0	4.1–7.8	9.1	6.9–11.2	2.3	1.0–3.7	13.5	10.8–16.2
Rank								
Commissioned officer	6.9	4.7–9.1	10.3	7.4–13.2	3.9	2.1–5.8	16.6	13.0–20.1
Non-commissioned officer	8.3	6.7–9.9	14.9	12.5–17.4	3.8	2.6–5.0	19.7	17.0–22.4
Other ranks	13.3	6.4–20.1	18.1	9.9–26.4	8.1	4.1–12.0	29.5	20.4–38.5
ADF deployment								
Yes	9.6	6.4–12.9	15.2	11.7–18.8	4.4	3.2–5.7	20.8	17.1–24.6
No	9.3	6.4–12.2	14.2	9.0–19.3	6.4	3.4–9.4	23.9	18.2–29.7

Table 5 shows the demographic correlates of any affective, anxiety, and alcohol disorder; and any mental disorder. ADF females were at statistically increased odds of meeting criteria for an anxiety disorder (OR=1.56, 95% CI 1.11, 2.19), but were significantly less likely to report an alcohol disorder (OR=0.36, 95% CI 0.18, 0.75) compared to males. Air Force personnel reported the lowest rates of all disorder types. Statistically, Army personnel were more likely to meet criteria for all categories of disorder: any affective (OR=1.56, 95% CI 1.02, 2.40), any anxiety (OR=1.65, 95% CI 1.10, 2.47), any alcohol (OR=2.53, 95% CI 1.26, 5.07), and any disorder (OR=1.77, 95% CI 1.25, 2.49) compared to the Air Force. Alcohol disorder was most prevalent in the Navy, with Navy personnel being statistically more likely to report an alcohol disorder (OR=3.57, 95% CI 1.67, 7.63) and any mental disorder (OR=1.71, 95% CI 1.21, 2.40) compared to the Air Force. ADF personnel in the lower ranks (other ranks) reported the highest rates of all disorder types. Compared to commissioned officers, ADF personnel in the other ranks (OR=1.91, 95% CI 1.01, 3.61) and non-commissioned officers (OR=1.50, 95% CI 1.02, 2.21) were statistically more likely to meet criteria for an anxiety disorder, but not an affective or alcohol disorder.

**Table 5:** Adjusted^a^ odds of 12-month ICD-10 disorder among Australian Defence Force Personnel sub-groups

	Any affective disorder		Any anxiety disorder		Any alcohol disorder		Any disorder	
				
Characteristic	OR	CI	OR	CI	OR	CI	OR	CI
Sex								
Female	1.18	0.81–1.72	1.56	1.11–2.19	0.36	0.18–0.75	1.21	0.90–1.62
Male^b^	1.0	–	1.0	–	1.0	–	1.0	–
Service								
Navy	1.54	0.95–2.49	1.26	0.87–1.82	3.57	1.67–7.63	1.71	1.21–2.40
Army	1.56	1.02–2.40	1.65	1.10–2.47	2.53	1.26–5.07	1.77	1.25–2.49
Air Force^b^	1.0	–	1.0	–	1.0	–	1.0	–
Rank								
Commissioned officer^b^	1.0	–	1.0	–	1.0	–	1.0	–
Non-commissioned officer	1.19	0.78–1.82	1.50	1.02–2.21	0.93	0.51–1.71	1.23	0.88–1.70
Other ranks	2.04	0.97–4.26	1.91	1.01–3.61	1.71	0.86–3.39	1.92	1.16–3.18
ADF deployment								
Yes	1.17	0.64–2.13	1.12	0.64–1.96	0.70	0.40–1.24	0.89	0.58–1.36
No^b^	1.0	–	1.0	–	1.0	–	1.0	–

OR, odds ratio; CI, 95% confidence interval.

a All characteristics were entered simultaneously into a multivariate logistic regression.

b Reference category for measure of association.

The majority of the ADF population had been on operational deployment at least once (62%), with 43% having deployed multiple times, and 37% having deployed to the MEAO (Afghanistan or Iraq) at some point in the ADF career. The prevalence of all disorder categories was very similar between the ever-deployed group and the never-deployed group, and not statistically different. A further analysis of the type of deployment (categorised as war-like and non-warlike) also did not reveal any significant differences in disorder prevalence.

## Discussion

One in five (22%) of the ADF population had experienced a mental disorder in the previous 12 months; 9.5% met criteria for an ICD-10 affective disorder, 14.8% met criteria for an ICD-10 anxiety disorder, and 5.2% met criteria for an ICD-10 alcohol disorder. This level of mental illness in the ADF suggests that despite the fact that the ADF is a selected and trained population that generally has better access to health care, this population bares a burden of psychiatric morbidity related to the nature of their work. The most comparable study is the investigation of the Canadian Forces, where a stratified sample was interviewed using an earlier version of the CIDI (Sareen et al., 2007). The study revealed that 14.9% of the Canadian Forces had a mental disorder, which is not directly comparable because only 6 axis one disorders were assessed in contrast to the 13 disorders assessed in the ADF. Also the two studies used different diagnostic criteria to analyse the data, with the Canadians using the *Diagnostic and Statistical Manual of Mental Disorders*—4th edition (DSM-IV) diagnostic criteria, and the current study using the ICD-10 criteria to allow comparison with the Australian population mental health prevalence rates.

Neither the United Kingdom nor the United States has yet conducted a prevalence study in their defence forces using a structured diagnostic interview. Large scale population-based studies of the UK forces conducted by the King's Centre for Military Health Research, used the GHQ-12 to denote the presence of a common mental disorder and reported a weighted prevalence of 20% (Hotopf et al., 2006). Iverson and colleagues used a clinical based telephone interview to administer the PC-PTSD and PHQ. They reported a weighted prevalence of 28.9% with any PHQ diagnosis or PTSD, 4.8% with probable PTSD, 11.0% with any depressive syndrome (3.7% with major depressive syndrome), 4.5% with any anxiety syndrome, and 18% with alcohol abuse (Iversen et al., 2009). Riddle et al. (2007), using US Millennium cohort data report a prevalence of 18.3% of any PHQ diagnosis or PTSD using the PCL. The direct comparability of these rates with ADF rates however, remain unclear because of the different mental health measures and survey methodology used, but are generally similar in magnitude (Riddle et al., 2007).

Our results suggest that the biggest challenge facing the ADF is anxiety disorders, a finding that is probably accounted for because of the inclusion of simple phobia, obsessive-compulsive disorder, and panic disorder when these disorders are often not assessed. As a consequence, in studies of other military cohorts, alcohol disorders and major depression appear to be the most prevalent disorder types (Iversen et al., 2009; Riddle et al., 2007; Sareen et al., 2007). The low prevalence of alcohol disorder in the ADF is consistent with ADF post-deployment screening data for personnel returning from deployment to the MEAO which showed that the majority of personnel reported in the low risk Zone 1 (83.4%) on the AUDIT (Benassi & Steele, 2011). In this data less than 1% reported drinking at harmful (0.7%) or dependent (0.4%) levels in the reintegration phase (3–6 months) following return from deployment to the MEAO. In interpreting these findings it is important to distinguish alcohol disorder from alcohol consumption. Excessive alcohol use is common in military personnel (Bray, Brown, & Williams, 2013). Studies of UK and US personnel for example have reported alcohol abuse in 12–13% of personnel with binge drinking rates being as high as 43% in US military personnel (Riddle et al., 2007; Ryan et al., 2007; Stahre, Brewer, Fonseca, & Naimi, 2009). In the Australian Gulf War Veterans study, 25.7% exhibited AUDIT caseness using their self-determined optimal cut-off score of 10 (McKenzie et al., 2006). In contrast, studies of diagnosable alcohol disorders are rare. To our knowledge, prior to the current study, the only interview-based study of alcohol disorder in a currently serving defence force to date, examined the Canadian Forces and demonstrated a prevalence of DSM-IV alcohol dependence of 4.8% (Sareen et al., 2007). In comparison, the prevalence of alcohol dependence (2.3%) found in the current study is slightly lower which may be in part a result of the difference between diagnostic criteria between DSM-IV and ICD-10. In a sample of first Gulf War Australian veterans, Ikin and colleagues (Ikin et al., 2004) reported 4.3% of (predominantly Navy) Gulf War veterans and 2.5% of a military comparison group had DSM-IV alcohol dependence or abuse using the CIDI. Despite the fact that this study comprised mostly naval personnel, these results are comparable to those reported in the current study and are therefore likely to represent an accurate picture of alcohol disorder in the Australian military.

Overall, Army personnel and personnel in the lower ranks were identified as being at particular risk of reporting a mental disorder. Females were at an increased risk of meeting diagnostic criteria for an anxiety disorder whereas males were statistically more likely to report an alcohol disorder. This pattern of socio-demographic risk factors has been identified previously (Riddle et al., 2007).

In this study, the 62% of ADF members who had been on operational deployment were not at an increased risk of developing an anxiety, affective, or alcohol disorder compared to those who had never deployed. To date, the cohort of Iraq and Afghanistan veterans (*N=*18,625) have not been compared to those not deployed to these theatres of operations (*N=*31,423). A comparison of the rates of disorder in this cohort with other international studies will be important to examine the extent to which this cohort has had an impact on the overall rates of disorder in the ADF. However, this finding about the impact of deployment remained, regardless of deployment type (warlike/non-warlike). This absence of a deployment related effect has been reported elsewhere, particularly in UK veterans returning from the 2003 war in Iraq (Hotopf et al., 2006) and therefore may represent a true underlying effect. Alternatively, ADF members in the non-deployed group may have had increased rates of other lifetime trauma such as childhood adversity and other accidents (Jones et al., 2013), may overall be lower in rank, may have been less able to deploy because of ongoing physical or mental health problems (a “healthy warrior effect” in deployed personnel) (Hotopf et al., 2006; Richardson, Frueh, & Acierno, 2010), or have been exposed to other stressful aspects of military service which do not fit the operational classification (Jones et al., 2013) (for example, border patrol), all of which have been reported in the literature to increase risk of mental health problems in military personnel. Finally, the proportion of deployed personnel exposed to combat, a known risk factor for mental health disorder following deployment (Sareen et al., 2007), may be small, reducing the likelihood of a significant effect. Additional research on this cohort needs to examine these factors in more detail.

This study has several important strengths. First and most importantly, this study is a representative study of mental disorder prevalence in an entire military population assessed using a structured diagnostic interview. All currently serving ADF members were invited to participate in the study, removing the selection bias inherent in other study designs (Riddle et al., 2007). Demographic information obtained from military records for *all* currently serving members (including non-responders) has allowed weighting of data to represent the entire ADF population (50,049 members). These data will produce accurate and unbiased mental disorder ADF population prevalence estimates.

The study constitutes a representative sample from all three service types (Navy, Army, and Air Force), both males and females (who were oversampled), deployed and non-deployed personnel, as well as personnel from the Special Forces. The sample size affords the statistical power to assess the relative contributions of gender, service, deployment, and rank (among other factors) to the prediction of mental disorder. To our knowledge, only one other military study is comparable in design (Belik, Stein, Asmundson, & Sareen, 2010; Sareen et al., 2007).

The two-phase design and stratification strategy used reduces the possibility of error in making prevalence estimates by focussing the diagnostic assessment on the respondents most likely to have a disorder. Additionally, because the interviewees were drawn from the large proportion of the ADF population who provided responses to the Phase 1 questionnaire, the potential for sampling error was further reduced. Furthermore, the use of diagnostic interviews reduces the bias in response validity associated with self-report surveys.

The use of the “gold standard” CIDI to examine rates of mental disorder provides prevalence estimates using ICD-10 criteria. This enables a direct comparison with estimates obtained from the 2007 Australian National Mental Health and Wellbeing Study (to be published in detail in a further publication). Furthermore, assessment of lifetime prevalence allows us to both distinguish between the proportion of ADF members who have experienced a disorder up to the date of the interview (lifetime prevalence) from the proportion of ADF members who will develop each disorder over their lifetime (projected lifetime risk) (Kessler et al., 2007) as well as provide valuable insight into the degree of pre-enlistment disorder that exists in the ADF and how this influences future psychological health in this population. This is beyond the scope of the current paper but will also be addressed in future publications on this dataset.

This is the first study to investigate the prevalence of mental disorder in the ADF in addition to a range of other important occupational and help-seeking-related factors. This allows an investigation into the complex interaction between risk and protective factors that have an impact on the psychological health of this population.

Finally, study results have important implications for prevention and early intervention programs, service delivery and treatment, and surveillance and detection, and are applicable to all military populations worldwide.

### Limitations

Several limitations could affect the interpretation of our data. Detailed questions relating to specific deployments (e.g., exposure to trauma and subsequent disorder prevalence associated with deployments) are not included. This limits the capacity to examine the contributions of specific aspects of deployment on the type and prevalence of disorder. However, we assess broader-level deployment factors including when, where, and how many times members deploy, including warlike and peacekeeping operations.

Like many occupational cohort studies that assess participants of various ages and service lengths, our data may be limited by the “healthy warrior effect”(Haley, 1998) where ADF members with poorer mental health are more likely to leave the Defence Force earlier either voluntarily or by involuntary discharge and are less likely to deploy. Thus, all associations and prevalence rates obtained in this report should be viewed in light of this fact.

## Conclusions

The ADF MHPWS is the first study to use a two-stage design in combination with a diagnostic interview to determine the rates of mental disorder in an entire military population, excluding reservists. Using this two-stage design, one in five ADF members met criteria for a diagnosable disorder in the past 12 months. This rate was consistent across ADF members who had deployed and those that had not. Future research should consider the broad impact of military service beyond deployment in order to provide better estimates of the true health effects of military service.

## Footnotes

1 As at 11th December 2009, identified through military records.
